# Get-Up-And-Go Hospitals and Hotels

**Published:** 1990-06

**Authors:** J. D. Davies


### Correspondents' column

**Get-up-and-go Hospitals and Hotels**

Don't get me wrong, I am all gratitude as a patient, a congenital insomniac, and love things going on at night.

We live in times of financial entrepreneurism. Thatcherism rules. At the moment there is little hint of extension into the tourist industry on the part of hospitals. This could be changed a bit. John Burton delicately touched on the subject in this journal a few months ago. It all depends on a different approach to tourism.

My father always possessed a nice sense of justice. He lived and worked as the local general practitioner in a quiet suburban road close to Kew Gardens. He felt that holidays should give a decent complementary balance to everyday life. He wished family holidays to have a different character from day-to-day home life. Although he enjoyed France immensely, and to France we certainly went, he did not relish long journeys in search of a southern sun. He had seen too much of that in the Western desert during the early 1940s. No, the Pas de Calais had much in its favour. Close to, and not too hot.

Nowadays long-distance travel is commonplace and it might be thought that the Pas de Calais or even Normandy have little to offer in the way of excitement. Shortly after the war, though, it was after all still 'abroad'.

In general I think that my mother was quite happy with such arrangements. However, I have omitted to mention one peculiarity in taste, which on mature reflection evidently dictated my father's choice of hotel. This, I think, stemmed from the tranquility of our own house in the London suburbs. Its complementary opposite would obviously have been in the midst of gear-changing lorry traffic, preferably on a hill, accompanied by much to-ing and fro-ing on the pavement.

My father, once left in charge of the affair was a master at finding hotels satisfying such criteria. Curiously few guide-books have ever promoted their virtues; indeed that wretched Red Michelin Guide persists to this day in taking a contrary view. One despairs about such prejudice. These sadly neglected noisy hotels often stand on cross roads close to the town centre (ignore anywhere where by-passes have been built; a most unhealthy trend). Their walls invariably excel in echoing the deep-throated roar of late-night heavy diesel-engined lorries. Police and fire engine klaxons are food and drink; windows were best permanently jammed open, or at least too warped to close properly—certainly no double glazing is allowable. To the conventional admirer of these hotels, a room at the front is especially desirable for the full frontal with the road traffic. It ought, I suppose, to be said tactfully that my mother proved occasionally less than a complete convert to this way of holidaymaking. Perhaps she didn't know how far in advance of his era my father was.

None the less, at these times, the truly dedicated expert in noisy hotels may resort to subterfuge. One simple such exercise is the discovery of a hotel with rear rooms giving onto a mainline railway track that carries the early morning expresses to Paris, preferably picking up local mail or better still—milk. There is a very satisfying ring to milk cannisters. Cattle markets or late-night fairs with dodgem cars, not to mention substantially built belfreys are other variants on this theme (agreed you need to know the best day of week for these lesser establishments in order to reveal their true worth). Surprisingly often, even suspicious wives fail to pick out these ancillary attactions when they are inconspicuously sited at the back of a hotel. A little tree screening can also help matters no end in this respect.

Not very long ago—many years after these holidays in the Pas de Calais—I managed to have what is called in polite circles a 'temporary suspension of service', and had to be admitted rather smartish to the BRI. Once the initial drama was over and things medical began to pall, wider prospects begin to intrude.

I was inadvertently reminded at the time of my father's idiosyncratic bent in the selection of hotels for holidaying. I really do feel sorry for him in that he never worked at the Bristol Royal Infirmary. From recent experience, I am certain that he would have dropped even the idea of crossing the Channel. For an in-patient, vainly attempting to sleep, the traffic noise is fantastic—a rare jewel of perfection, particularly in the wards facing the Marlborough Street chasm. Just a brief dull interval of comparative quiet between 2 and 4 o'clock in the early morning. Apart from that there is a continuously changing growl or roar of cars and buses, punctuated from time to time by the fluorescent chirp of pedestrian crossings, open-top reggae or the healthy scream from a decently unsilenced motorbike. Choose your season well, and British Telecom may be having a go with man-sized trenching.

There is thus no need to go all the way to France. If it were possible to change the ward television channel, you could easily be in Wissant or Rouen. I am sure there are tourist possibilities here. It does seem a shame to have to go to all the effort of being ill in order to savour our special Bristol amenities.

Maybe it could all be part of an independent Trust. High tech medicine is very expensive. A proper 'Hotel Section', by which I do not mean NHS 'Hotel Services', might bring in a better rate of return. Look at the waste of opportunities with the old French Hospital or the Soho for Women in London. Surely the provinces can do better. Just ask me, I've always fancied a public relations job.

J. D. Davies

